# Inter- and Intra-Observer Variability of the AMADEUS Tool for Osteochondral Lesions of the Talus

**DOI:** 10.3390/jpm14070749

**Published:** 2024-07-15

**Authors:** Konstantinos Tsikopoulos, Jenn Wong, Moustafa Mahmoud, Vasileios Lampridis, Perry Liu, Radoslaw Rippel, Alisdair Felstead

**Affiliations:** 1 Orthopaedic Department, Portsmouth Hospitals University NHS Trust, Portsmouth PO6 3LY, UKperry.liu@porthosp.nhs.uk (P.L.);; 2 Radiology Department, Portsmouth Hospitals University NHS Trust, Portsmouth PO6 3LY, UKradoslaw.rippel@porthosp.nhs.uk (R.R.); 3 Orthopaedic Department, Liverpool University Hospitals, Liverpool L9 7AL, UK; vlampridis@hotmail.com

**Keywords:** cartilage defects, inter-observer variability, intra-observer variability, osteochondral lesions, bone marrow stimulation, AMADEUS scoring system

## Abstract

Background: Managing osteochondral cartilage defects (OCDs) of the talus is a common daily challenge in orthopaedics as they predispose patients to further cartilage damage and progression to osteoarthritis. Therefore, the implementation of a reliable tool to quantify the amount of cartilage damage that is present is of the essence. Methods: We retrospectively identified 15 adult patients diagnosed with uncontained OCDs of the talus measuring <150 mm^2^, which were treated arthroscopically with bone marrow stimulation. Five independent assessors evaluated the pre-operative MRI scans with the AMADEUS scoring system (i.e., MR-based pre-operative assessment system) and the intra-/inter-observer variability was then calculated by means of the intraclass correlation coefficients (ICC) and Kappa (κ) statistics, respectively. In addition, the correlation between the mean AMADEUS scores and pre-operative self-reported outcomes as measured by the Manchester–Oxford foot questionnaire (MOxFQ) was assessed. Results: The mean ICC and the κ statistic were 0.82 (95% CI [0.71, 0.94]) and 0.42 (95% CI [0.25, 0.59]). The Pearson correlation coefficient was found to be r = −0.618 (*p* = 0.014). Conclusions: The AMADEUS tool, which was originally designed to quantify knee osteochondral defect severity prior to cartilage repair surgery, demonstrated good reliability and moderate inter-observer variability for small OCDs of the talar shoulder. Given the strong negative correlation between the AMADEUS tool and pre-operative clinical scores, this tool could be implemented in clinical practise to reliably quantify the extent of the osteochondral defects of the talus.

## 1. Introduction

Cartilage defects of the talus represent a diagnostic and therapeutic challenge [1] as they predispose patients to further cartilage damage and progression to osteoarthritis. Therefore, optimising the pre-operative assessment of those patients by implementing a reliable diagnostic tool to accurately quantify the amount of cartilage damage that is present and predict post-operative outcomes is essential.

The treatment options for those cartilage defects vary depending on the size and stability of the lesion [1]. Bone marrow stimulation (BMS) demonstrates an 85% success rate for lesions smaller than 15 mm or shallower than 7 mm [2]. It is worth noting that, following bone marrow stimulation, fibrocartilage forms which predominantly consist of Type 1 collagen, are thus less durable compared to normal hyaline (Type 2 collagen) cartilage. As a result, a recent systematic review of level 4 evidence has demonstrated that, despite the fact that the mid-term (i.e., average 48-month follow-up) clinical results following BMS for the treatment of primary OCDs are satisfactory, radiological deterioration is more often than not documented [3]. With a satisfactory complication profile and a mid-term reoperation rate of 6.0%, BMS remains the standard of care treatment after failed conservative treatment for the above groups of patients [3].

Following the original radiographic classification of OCDs from Bernt and Harty in 1959, which was based on the integrity of the osteochondral fragment, additional MRI-based systems were proposed. Nevertheless, the need for developing a reliable and clinically relevant system remains. The Area Measurement and Depth and Underlying Structures (AMADEUS) scoring system is currently used for the pre-operative assessment of osteochondral defects in the knee. It is a comprehensive three-part classification system that rates (1) the cartilage defect area; (2) the depth of the lesion; and (3) the underlying structures with the associated bone marrow oedema and osseous defects. Not only does it allow for a reliable assessment of the severity of the defects, but also it is clinically applicable in daily practise [4].

We wish to highlight the importance of utilising an easily applicable MRI-based diagnostic tool which could facilitate interdisciplinary communication between orthopaedic surgeons and clinical radiologists, thus supporting decision making and communication with patients as well. The purpose of this study was to determine the intra- and inter-observer variability of a tool that was originally designed for knee pathology (i.e., the AMADEUS grading system) in adult patients undergoing arthroscopic bone marrow stimulation for talar OCDs. In addition, we sought to assess the potential correlation between pre-operative self-reported outcomes and AMADEUS scores.

## 2. Materials and Methods

The study was registered locally at our institution (ID 5751) and Guidelines for Reporting Reliability and Agreement Studies (GRRAS) were followed [5].

We included adult male and female patients diagnosed with symptomatic uncontained (i.e., shoulder) talar OCDs measuring <150 mm^2^ which were subsequently treated with BMS. To define an osteochondral lesion, its largest diameters on the coronal, sagittal and axial planes were considered [6]. For a patient to qualify for inclusion, a minimum post-operative follow-up of 9 months was required with the corresponding self-administered questionnaire being filled in. Ankles with bipolar osteochondral lesions or osteoarthritis were excluded. Also, patients with associated ankle pathology undergoing concomitant procedures such as lateral ankle ligament stabilisation that could affect clinical outcomes were ruled out.

Each patient underwent a pre-operative 3 T or 1.5 T MR examination with the appropriate sequences [4]. The MR images were subsequently transferred to the local hospital picture archiving and communication system (PACS). Five independent assessors (two senior radiologists and three orthopaedic doctors) evaluated the pre-operative MRI scans with the AMADEUS scoring system [4]. As per this tool, three metrics are used to describe a focal chondral or an osteochondral lesion. To be more exact, the points for the three metrics and the addendum add up to give a total score of 100 (best score) to 0 (worst score). The three metrics are as follows: (1) Description of the defect area in one plane multiplied by the diameter in the other plane. (2) Description of the defect morphology/depth. (3) Description of the condition of the underlying structures (subchondral bone; A, B or C) and bone marrow oedema-like lesions (BME; E) as an addendum.

### 2.1. Outcome Assessment

The primary outcome of the study was to assess the inter- and intra-observer variability of the AMADEUS scoring system for osteochondral lesions of the talus. To achieve this objective, each of the assessors evaluated the scans twice (two weeks apart) and the average reliability was subsequently calculated [7]. To elaborate, the intra-/inter-observer variability was then calculated by means of the intra-class correlation coefficients (ICC) and Kappa (κ) statistics, respectively [7,8]. The following classification was implemented:

- Intra-class correlation (ICC) < 0.5 denoted poor reliability;
- 0.5 < ICC < 0.75 showed moderate reliability;
- 0.75 < ICC < 0.9 indicated good reliability;
- ICC > 0.9 showed excellent reliability.

For the inter-observer reliability evaluation, the opinions provided by different assessors for each case were compared and the overall agreement was then calculated according to the Landis and Koch grading system [9]. The κ values were classified as follows:

- κ < 0.2 denoted slight agreement;
- 0.2 < κ < 0.4 showed fair agreement;
- 0.4 < κ < 0.6 indicated moderate agreement;
- 0.6 < κ < 0.8 showed substantial agreement;
- κ > 0.8 showed perfect agreement.

The secondary outcome included the assessment of the statistical correlation between the pre-operative clinical scores and the mean AMADEUS scores. The clinical outcome data were collected from an electronic database maintained at our institution. If any additional information was required, the participants were contacted, and the missing data were gathered.

### 2.2. Statistics

First of all, the sample size was calculated according to published guidelines for reliability studies [10]. With an expected reliability of 0.8, a confidence level of 95% CI and a power of 80%, a minimum number of 14 patients was calculated based upon the primary outcome of the current study. Of note, a *p* value of <0.05 indicated statistical significance.

The original percentage AMADEUS scores were transformed to the AMADEUS grade ranging from I to IV (I = best grade, IV = worst grade) and the pre-operative Manchester–Oxford Foot Questionnaire (MOxFQ) outcomes were compared with parametric statistical tests using the SPSS 27 software (SPSS, Chicago, IL, USA). In addition, the pre-operative mean MOxFQ scores of the available AMADEUS stages were compared to each other.

The linear correlation between the mean AMADEUS scores and pre-operative MOxFQ scores was assessed by means of the Pearson correlation coefficient and classified as shown below [11], with a *p* value of less than 0.05 demonstrating statistical significance:

- Correlation < 0.20: very weak;
- Correlation 0.20–0.39: weak;
- Correlation 0.40–0.59: moderate;
- Correlation 0.60–0.79: strong;
- Correlation > 0.80: very strong.

## 3. Results

### 3.1. Demographics

A total of 15 patients were recruited according to our pre-defined selection criteria. The mean age of the participants was 43.2 years (SD = 14.3), with four of the participants being females and ten males (Table 1).

### 3.2. Reliability and Agreement Assessment

#### 3.2.1. Intra-Class Correlation

The normality test revealed the normal distribution and the mean ICC was found to be 0.8226 (95% CI 0.71 to 0.94). It should be noted that no statistical significance was noted when the ICC data were compared between the radiologists and the orthopaedic surgeons (mean difference was −0.1; 95% CI −0.353 to 0.146; *p* = 0.277).

#### 3.2.2. Percentage Agreement and Inter-Class Correlation

The percentage agreement between the MRI scan assessors was 56.67%. After adjusting for chance, the κ value was found to be 0.42 (95% CI [0.25, 0.59]).

### 3.3. Secondary Outcome Assessment

The correlation between the pre-operative MOxFQ and mean AMADEUS scores was found to be r = −0.618, *p* = 0.014.

## 4. Discussion

In this paper, we assessed the inter- and intra-observer variability of the AMADEUS grading system, which was originally designed for knee osteochondral lesions, for OCDs of the talar shoulder. To minimise heterogeneity in this report, only uncontained (shoulder) lesions (that is, the absence of a peripheral cartilage border on one side with the loss of the medial or lateral articular buttress) were considered, as there is evidence to suggest that the presence of contained (non-shoulder) lesions exerts a positive impact on clinical results in patients with talar OCDs [12]. Of note, only lesions measuring less than 150 mm^2^ were included, as clinical outcomes worsen significantly if BMS is implemented for defects beyond this critical defect area [13]. We showed that there is satisfactory reliability and moderate agreement when using the above MRI-based tool for talar OCDs. Those findings remained robust when we accounted for the subspeciality of the assessors (i.e., radiologists vs. orthopaedic surgeons). Of note, a strong negative correlation between the pre-operative MOxFH and mean AMADEUS scores was documented. In other words, there was a positive linear correlation between the pre-operative clinical and radiological outcomes.

### 4.1. Selecting the Appropriate Diagnostic Imaging Tool Pre-Operatively

Despite the fact that CT arthrography demonstrates satisfactory sensitivity and specificity, that is, 99% and 81% [14], respectively, it cannot detect chondromalacia. By contrast, MRI can reliably assess the stability of the osteochondral fragment [15] and evaluate all cartilage layers (that is, the surface, the cartilage substance and the subchondral bone) [16]. To be more exact, MRI has a sensitivity and specificity of 96% for diagnosing OCDs [17] and is currently considered to be the most appropriate advanced imaging method for this condition. Consequently, CT arthrography is only recommended if in the presence of contraindications for MRI scans and when further diagnostic information is required [16].

### 4.2. AMADEUS and Other MRI-Based Scoring Systems

Many MRI-based classification systems have been presented over the last few decades; however, their clinical relevance is either questionable or has yet to be determined. A commonly used classification system is that reported by Hepple et al. [15], which accounts for the presence of subchondral oedema [15,18]. In this study, we selected the AMADEUS tool due to the fact that the recent literature stemming from knee surgery has shown that it allows for the reliable severity encoding, scoring and grading of osteochondral defects. Furthermore, it is easy to use in the clinical setting as there are clear diagrams and instructions to explain the descriptors. In addition, no familiarity with the talus is specifically required. We highlight that, unlike the previously reported classification systems, the AMADEUS tool considers all the fragment instability signs in a native MRI scan, which include signal changes around the OCD, subchondral changes and focal cartilage defects, all of which can affect treatment [19]. In addition, accounting for bone marrow oedema lesions in the talar dome is of clinical importance as the cartilage condition of the osteochondral fragment may be predicted [20]. To our knowledge, there is no other scoring system in the literature demonstrating higher reliability and inter-observer variability when compared to the AMADEUS tool for talar OCDs.

### 4.3. Prognostic Factors for the Operative Treatment of OCDs of the Talus

We wish to draw the reader’s attention to the fact that there are several other clinical factors that can potentially affect post-operative results substantially. For example, other positive factors could be contained and anterolateral lesions [12,21]. Conversely, negative indicators include medial talar lesions [22], older age (>33–40 years old) [23] and higher BMI, a longer duration of symptoms, an association with a traumatic event and the presence of osteophytes [24]. For the above reasons, we claim that an investigation of the correlation between the AMADEUS scores and the post-operative outcomes could have been inappropriate and it was avoided in the current paper.

### 4.4. Study Limitations and Implications for Future Research

We recognise that this paper has a few limitations. First, the sample size was small to allow for robust conclusions on the self-reported post-operative clinical outcomes. This was because stringent inclusion criteria were implemented to achieve homogeneity in the results and due to the fact that the power calculation was based upon the reliability assessment of the AMADEUS tool. Second, we only investigated patients treated with bone marrow stimulation for small osteochondral defects of the talus. Therefore, more original studies are needed to assess the correlation between the AMADEUS scoring system and clinical outcomes in patients with large defects managed with different surgical techniques (e.g., AMIC, ACI, OATS). Third, we have been unable to perform a correlation analysis to compare the pre-operative scores with the arthroscopic findings, which would have improved the credibility of our study. Fourth, the presence of loose fragments in the ankle joint is an important parameter potentially affecting clinical outcomes in OCDs. Therefore, we advocate that future authors could incorporate it into a modified AMADEUS scoring system. Likewise, the lesion-containment factor and the underlying pathology could be considered, as these parameters do affect treatment outcomes. Last but not least, we claim that the implementation of a reliable MRI-based diagnostic tool in future foot and ankle clinical practise is of the utmost importance for the frequently encountered clinical problem of osteochondral lesions of the talus.

## 5. Conclusions

In conclusion, we advocate that the AMADEUS tool demonstrates satisfactory reliability and moderate inter-observer variability and therefore it could be incorporated into the pre-operative assessment of patients who are due to undergo bone marrow stimulation for small osteochondral defects of the shoulder of the talus measuring <150 mm^2^. Furthermore, there was a strong correlation between the pre-operative clinical and radiological outcomes, which reflects the clinical usefulness of this tool for talar dome lesions. However, we wish to highlight the fact that further research on this topic is needed to reach more robust clinical conclusions spanning the whole spectrum of the osteochondral defects of the talus.

## Figures and Tables

**Table 1:** Baseline demographic characteristics of the included patients.

Patient	Sex	Age	Body Mass Index (kg/m^2^)	Laterality	Talar Dome Location	Mean AMADEUS Score	MOxFQ Baseline Score
1	Female	72	31.1	Right	Medial	56	42/64
2	Male	57	36.5	Left	Medial	39	47/64
3	Female	53	30.4	Left	Medial	69	42/64
4	Male	40	17.4	Left	Medial	59	44/64
5	Female	49	29.4	Left	Medial	62	41/64
6	Male	46	26.9	Right	Medial	83	31/64
7	Female	39	28.4	Right	Lateral	65	38/64
8	Male	40	35.3	Left	Lateral	64	42/64
9	Male	37	34.1	Right	Medial	36	45/64
10	Male	35	21.8	Left	Medial	52	44/64
11	Female	18	32	Right	Lateral	77	43/64
12	Male	39	30.1	Right	Lateral	65	41/64
13	Male	45	31.4	Right	Lateral	77	37/64
14	Male	60	31	Right	Medial	40	40/64
15	Female	18	21.3	Left	Lateral	64	33/64

MOxFQ = Manchester–Oxford Foot Questionnaire.

## Data Availability

The original contributions presented in the study are included in the article, further inquiries can be directed to the corresponding author.
